# Branched-chain amino acids catabolism and cancer progression: focus on therapeutic interventions

**DOI:** 10.3389/fonc.2023.1220638

**Published:** 2023-08-10

**Authors:** Er Xu, Bangju Ji, Ketao Jin, Yefeng Chen

**Affiliations:** ^1^ Department of Hospital Infection Management, Affiliated Hospital of Shaoxing University, Shaoxing, Zhejiang, China; ^2^ Department of Colorectal Surgery, Shaoxing People’s Hospital, Shaoxing, Zhejiang, China; ^3^ Department of Colorectal Surgery, Affiliated Jinhua Hospital, Zhejiang University School of Medicine, Jinhua, Zhejiang, China; ^4^ Department of Respiratory Medicine, Shaoxing People’s Hospital, Shaoxing, Zhejiang, China

**Keywords:** branched-chain amino acids, cancer, metabolism, catabolism, cancer therapy

## Abstract

Branched-chain amino acids (BCAAs), including valine, leucine, and isoleucine, are crucial amino acids with significant implications in tumorigenesis across various human malignancies. Studies have demonstrated that altered BCAA metabolism can influence tumor growth and progression. Increased levels of BCAAs have been associated with tumor growth inhibition, indicating their potential as anti-cancer agents. Conversely, a deficiency in BCAAs can promote tumor metastasis to different organs due to the disruptive effects of high BCAA concentrations on tumor cell migration and invasion. This disruption is associated with tumor cell adhesion, angiogenesis, metastasis, and invasion. Furthermore, BCAAs serve as nitrogen donors, contributing to synthesizing macromolecules such as proteins and nucleotides crucial for cancer cell growth. Consequently, BCAAs exhibit a dual role in cancer, and their effects on tumor growth or inhibition are contingent upon various conditions and concentrations. This review discusses these contrasting findings, providing valuable insights into BCAA-related therapeutic interventions and ultimately contributing to a better understanding of their potential role in cancer treatment.

## 1 Introduction

Cancer is one of the most critical problems related to human health, which affects millions of people worldwide every year and leads to numerous deaths (1). Studies have revealed that the metabolism of tumor and immune cells in the tumor microenvironment (TME) plays a crucial role in cancer development and antitumor immune responses (2). For instance, tumor cells utilize various metabolic pathways to produce biological macromolecules that support their energy and proliferation (3, 4). On the other hand, the concentration of nutrients in the TME affects the selection of the metabolic pathways and macromolecules by tumor cells to reach the optimum conditions for their growth and survival. Among the macromolecules used by tumor and immune cells, amino acids, glucose, and fatty acids are essential for producing energy and growth (5–7).

Recent studies have suggested that an imbalance in amino acids, including essential and non-essential ones, may contribute to the pathogenesis of various human disorders, including cancer (8). Among these amino acids, branched-chain amino acids (BCAAs), including leucine, isoleucine, and valine, are essential and cannot be synthesized by the human body. Therefore, including BCAA-rich proteins in the diet is necessary to support protein synthesis, increase albumin levels, and prevent proteolysis (9, 10). Interestingly, BCAAs have been found to inhibit tumor cell migration and metastasis by reducing the expression of N-cadherin (11). However, the role of BCAAs in cancer remains controversial, as some studies have indicated that the catabolism of these amino acids can enhance tumor growth and progression in a dose-dependent manner (12). As a result, targeting the enzymes involved in BCAA catabolism or utilizing BCAA-rich diets has emerged as potential therapeutic interventions (13). Nonetheless, due to the dual role of BCAAs in cancer, the implementation of these therapeutic approaches may present challenges and limitations (14).

This review delves into the role of BCAAs in cancer pathogenesis, and therapeutic interventions involving the inhibition of BCAA catabolism and the utilization of BCAA-rich diets for cancer treatment are summarized. Additionally, the achievements and limitations of these approaches are discussed, providing a comprehensive analysis of BCAA-related strategies in cancer treatment.

## 2 BCAA biology

The nine essential amino acids include valine, leucine, isoleucine, histidine, lysine, tryptophan, methionine, phenylalanine, and threonine (15). Among these crucial amino acids, BCAAs (leucine, isoleucine, and valine) have an aliphatic side chain and a branch composed of a central carbon bound to three or more carbon atoms. BCAAs are non-polar aliphatic amino acids consisting of a carboxylic acid group, an amino group, and an isopropyl group on the side chains. Valine (HO2CCH(NH2)CH(CH3)2) is an aliphatic and highly hydrophobic amino acid found in the interior of globular proteins. Valine preserves mental strength, promotes muscle growth, aids in tissue repair, and provides energy support as a glycogenic essential amino acid. Soy, fish, cheese, vegetables, and meats are rich sources of valine. L-Valine is one of the twenty proteinogenic amino acids encoded by GUC, GUU, GUA, and GUG codons (16).

Leucine (HO2CCH(NH2)CH2CH(CH3)2) is a hydrophobic amino acid encoded by UUA, UUG, CUC, CUU, CUG, and CUA codons. It is a critical component of astacin and ferritin subunits. Leucine deficiency is rare because it is available in several foods (17). Another BCAA, isoleucine (HO2CCH(NH2)CH(CH3)CH2CH3), is synthesized from pyruvate by bacteria that utilize leucine biosynthesis enzymes. Isoleucine is encoded by the AUU, AUC, and AUA codons and is found in seeds, nuts, meats, cheese, fish, and eggs (18).

BCAAs belong to the proteinogenic amino acids category (19). They play a role in several physiological and metabolic mechanisms, such as promoting protein synthesis and degradation in skeletal muscles and other tissues, hormone production, detoxification of nitrogenous wastes, wound healing, initiating signaling pathways, regulating gene expression, cell apoptosis and regeneration, insulin resistance, and glucose metabolism (20–22). BCAAs can promote glycogen sparing, and there is a close association between BCAAs, brain levels of tryptophan, and serotonin (23). The oxidation of BCAAs primarily occurs in skeletal muscles by specific enzymes, enhancing fatty acid oxidation and preventing obesity. However, other amino acids are catabolized in the liver (20). BCAAs also participate in immune system processes. It has been revealed that immune cells produce decarboxylase and dehydrogenase enzymes responsible for breaking down BCAAs, thereby improving lymphocyte growth and proliferation, dendritic cell (DC) maturation, and boosting cytotoxic T cell activity (24, 25).

Cells sense the availability of intracellular concentrations of BCAAs, such as leucine, to regulate ribosome biogenesis and protein synthesis. Phosphorylated sestrin2, by binding to gator2, negatively regulates the mammalian target of rapamycin complex 1 (mTORC1) protein kinase, which is involved in cell growth. It has been shown that sestrin is dephosphorylated after binding to leucine, reducing its inhibitory effect on gator2. As a result, the mTORC1 pathway is activated and leads to cell growth (26, 27). Another interesting interaction of leucine with mTORC1 is enhancing the acetylation of Raptor (a partner of mTORC1) by leucine-derived acetyl-CoA, which improves growth signals (28). Therefore, leucine can significantly promote cell growth by activating the mTORC1 pathway (29). Evidence suggests that uncontrolled activation of the mTOR pathway has been reported in several human cancers. Therefore, increasing the concentrations of BCAAs, especially leucine, may enhance the growth of tumor cells (30).

## 3 BCAAs catabolism

As mentioned before, BCAAs can be catabolized by enzymes, which affect the levels of leucine, isoleucine, and valine in an overlapping manner (29). Since leucine is abundant in proteins, the breakdown of exogenous proteins from foods or internal sources such as muscles releases a large amount of this amino acid (31). The final product of the oxidation of BCAAs varies depending on their type because studies have shown that the carbon precursors used for glucose production, which occurs independently of acetyl-CoA, are only produced by the catabolism of valine and isoleucine (29). Moreover, the production of carbon derived from BCAAs during their catabolism can provide the necessary fuel for the tricarboxylic acid (TCA) cycle and oxidative phosphorylation, supplying energy to the cells. Additionally, it has been shown that BCAA catabolism can facilitate the synthesis of other amino acids and nucleotides through the *de novo* pathway, as well as proteins, by affecting proteinogenic amino acids or enhancing nutritional cell signals. The breakdown of BCAAs produces metabolites that can potentially influence the epigenome. One notable example is the generation of acetyl-CoA from BCAA catabolism (29). Acetyl-CoA serves as a crucial supplier of acetyl groups for histone acetylation, thereby influencing the epigenetic characteristics of cells by affecting acetyl-CoA levels (32–34).

Transamination is an essential phenomenon in the catabolism of free BCAAs, carried out by the enzymes branched-chain amino acid transaminase 1 (BCAT1) and BCAT2. BCAAs can undergo transamination, and the transfer of nitrogen to α-ketoglutarate (α-KG) leads to the generation of glutamate and branched-chain keto acids (BCKAs), including ketovaline (α-ketoisovalerate), ketoleucine (α-ketoisocaproate), and ketoisoleucine (α-keto-β-methylvalerate). BCAT1 and BCAT2, both highly active reversible enzymes present in the cytosol and mitochondria, act upon all three BCAAs and their corresponding BCKAs. Interestingly, to regenerate α-KG and BCAA, BCAT1 and BCAT2 transfer nitrogen from glutamate back to a BCKA (29). Therefore, nitrogen can be replaced quickly between BCAAs, glutamate, α-KG, and BCKAs even in a minimum BCAAs catabolism state. A multimeric enzyme complex called BCKA dehydrogenase (BCKDH), which consists of E1, E2, and E3 subunits, catalyzes the oxidative decarboxylation of BCKAs to generate acyl-CoA derivatives, including isovaleryl-CoA, 2-methylbutyryl-CoA, and isobutyryl-CoA in various cells and tissues. These acyl-CoA derivatives are metabolized through distinct pathways. Specifically, valine catabolism yields propionyl-CoA, leucine generates acetyl-CoA and acetoacetate, and isoleucine produces acetyl-CoA and propionyl-CoA (35).

In mammals, the activity of BCKDH is irreversible. Additionally, the absence of enzymes synthesizing BCKAs from the *de novo* pathway makes it impossible to produce BCAAs in the body (36).

## 4 BCAA catabolism and cancer

**Figure 1:**
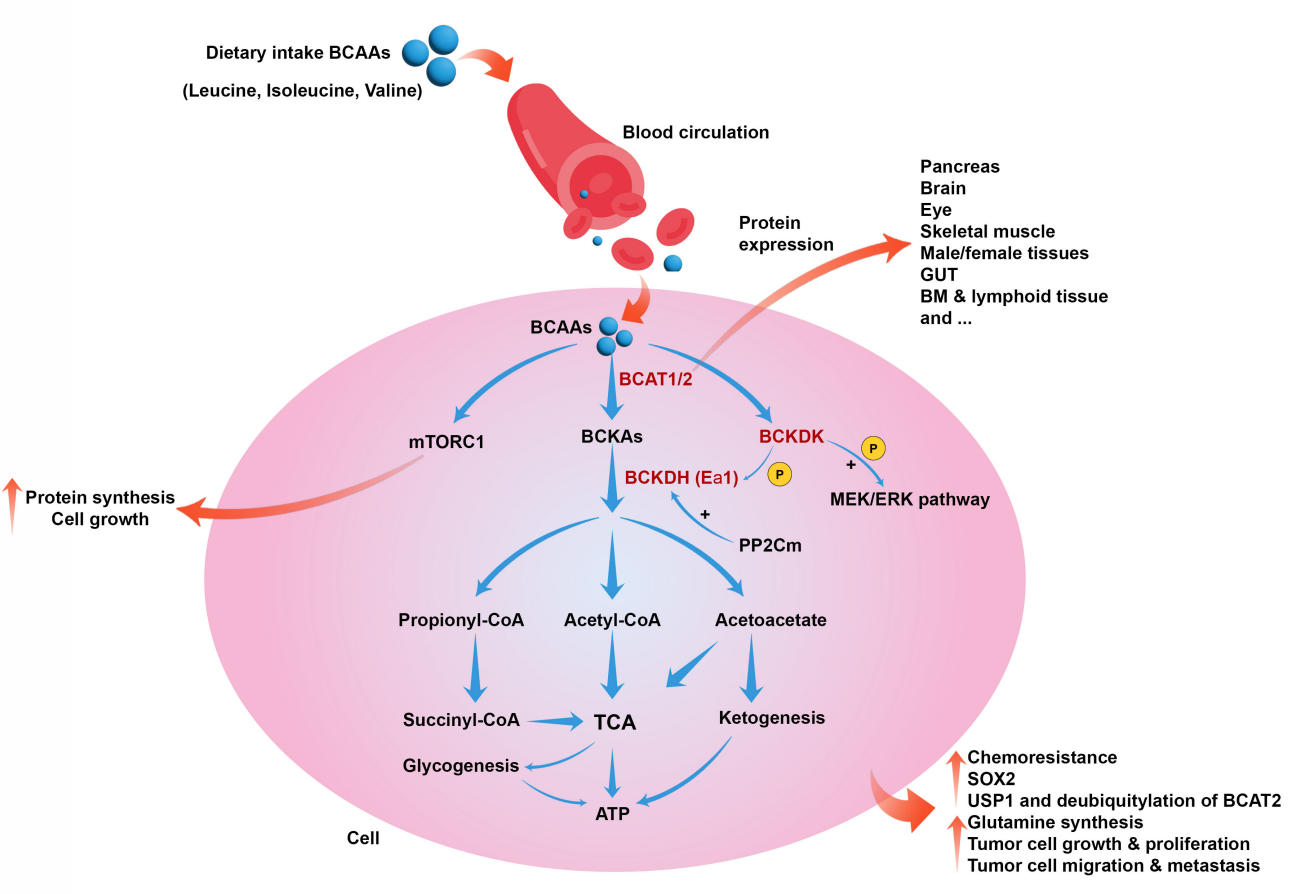
Role of BCAAs catabolism in cancer. BCAAs catabolism is induced by BCAT1, BCAT2, BCKDK, and BCKDH to produce metabolic intermediates such as acetyl-CoA, initiating the TCA cycle and producing energy. BCAAs also can induce mTORC1 and cell growth and proliferation. BCAAs catabolism can collectively lead to chemoresistance, upregulation of SOX2, induction of USP1 and deubiquitylation of BCAT2, glutamine synthesis, tumor cell growth, proliferation, migration, and metastasis.

Recent investigations show that several human disorders, such as cancer, can be associated with increased circulatory levels of BCAAs (**Figure 1**). Furthermore, whole-body protein turnover and dietary intake determine the plasma levels of BCAAs because the gut is responsible for BCAA absorption and their release into the blood circulation (37–39). In the conditions of need, BCAAs are released from proteins and catabolized in the tissues that express BCATs, such as skeletal muscles, leading to the production of BCKA and its release into the bloodstream. Due to the expression of BCKDH in the liver, circulatory BCKAs provide carbon for fatty acid synthesis, ketogenesis, and gluconeogenesis (40).

Systemic glucose metabolism can be affected by BCAAs via the regulation of insulin release and tissue sensitivity to this hormone (41, 42). On the other hand, insulin, through regulating the BCKDH enzyme in the liver, is involved in regulating BCAA catabolism. Therefore, impairment in the normal function of the pancreas, obesity, diabetes, and insulin resistance can affect BCAA breakdown in skeletal muscles, liver BCKA breakdown, and plasma concentrations of BCAAs (43). It has been reported that tumor cells may reprogram the systemic metabolism of BCAAs, leading to cancer progression (29, 44). Glutamine is one of the essential amino acids produced from BCAA catabolism and is a basic requirement for numerous tumor cells (35). Tumor cells use BCAAs as fuel, increasing the expression of BCAT1 and catabolizing them to provide glutamine. Studies have reported that the increase in BCAT1 enzyme levels is associated with tumor growth and proliferation in various cancers, and inhibiting it in certain cases, such as glioblastoma, leads to a decrease in tumor development (45–47).

Interestingly, in Hepatocellular carcinoma (HCC), only the levels of BCAT1/2 are upregulated, leading to chemoresistance, while BCKDH and other enzymes involved in BCAA catabolism are decreased (45, 48). In contrast, an investigation revealed that in breast cancer, along with BCAT1, the expression of BCKDHA and BCKDHB was upregulated, thus promoting tumor cell growth (49). BCAT1 also can induce the mTORC1 pathway to amplify mitochondrial biogenesis and generate ATP, providing energy and enhancing tumor cells’ growth and colony formation in breast cancer (49). Upregulation of BCAT1 is also reported in other human malignancies, such as ovarian cancer. An investigation on ovarian cancer cells showed that suppressing BCAT1 can downregulate involved genes in tumorigenesis and reduce ATP levels generated by the TCA cycle (50). BCAAs are used as a potential carbon source for the biogenesis of fatty acids to facilitate cell proliferation (12). In pancreatic ductal adenocarcinoma (PDAC), the concentrations of BCAA levels are significantly elevated, which is associated with tumor growth and development because BCAT2 is upregulated in PDAC, and the inhibition of this enzyme is associated with suppressing PDAC progression (51, 52).

Moreover, pancreatic stellate cells (PSCs) in PDAC regulate BCAA metabolism. Assessment of serum and tissue BCAAs in patients with PDAC revealed their levels significantly increased; however, adding BCAA to PDAC cell culture medium in several concentrations cannot affect tumor cell proliferation and migration. This study showed that following coculture of aPSCs with PDAC cells induced the proliferation of tumor cells by affecting the pattern of proteins involved in the BCAA degradation pathway (53).

A significant upregulation of BCKDK was reported in patients with colorectal cancer and was accompanied by an unpromising prognosis. Preclinical studies on colorectal cancer demonstrated that BCKDK activates MEK/ERK pathway, inducing tumorigenesis (54, 55).

Leucine is a ketogenic amino acid that can be imported from the circulation into the brain parenchyma (56). In this way, it can provide the metabolic requirements of cancer cells in brain tumors as an effective substrate and complement of glucose. Evidence shows that brain tumor cells can eliminate leucine from their milieu and augment their media with the metabolite 2-oxoisocaproate. Moreover, enriched media with a high leucine concentration produced 3-hydroxybutyrate and citrate, indicating metabolic reprogramming of tumor cells. In human tumor samples as well as cultured cancer cells, the expression of 3-methylcrotonyl-CoA carboxylase was detected. This enzyme is involved in the irreversible leucine catabolic processes (57). Accordingly, tumor cells can catabolize leucine and deliver its carbon atoms to the TCA cycle. In addition, leucine contributes to the production of lipids required for citrate to be withdrawn from the TCA cycle (57).

Serum metabolites in patients with non-small-cell lung cancer (NSCLC) and NSCLC cell cultures supernatant were examined, and findings showed that following upregulation of BCKDK, BCAA concentration significantly increased. At the same time, citrate levels were reduced in preoperative NSCLC patients than in healthy subjects. Furthermore, knockout of BCKDK inhibited tumor cell proliferation and induced apoptosis of NSCLC cells *ex vivo.* The oxidative phosphorylation and ROS levels increased, and glycolysis was repressed. These data indicated that BCKDK affects oxidative phosphorylation and glycolysis via regulating BCAA and citrate degradation, inducing NSCLC progression (58).

It has been revealed that liver cancer cells induce BCAA catabolism in the absence of glutamine, increasing carbon and nitrogen to generate nucleotide and enhancing the cell cycle and cell survival. Under glutamine deficiency conditions, O-linked-N-acetylglucosaminylation is increased, stabilizing mitochondrion protein phosphatase 1K (PPM1K) protein, as well as increasing BCKDHA dephosphorylation and BCAAs decomposition. An upregulation of BCKDHA and dephosphorylated BCKDHA induces tumorigenesis and is associated with poor prognosis in patients with HCC (59).

Analysis of genes involved in BCAA catabolism in pancreatic cancer showed that among dihydrolipoamide branched chain transacylase E2 (*DBT*), 4-aminobutyrate aminotransferase (*ABAT*), acetyl-CoA acetyltransferase 1 (*ACAT1*), *BCAT1*, and *BCAT2*, which were correlated to poor prognosis, *ABAT* and *BCAT2* were hub genes with satisfactory prognosis values. Moreover, the upregulation of *BCAT2* was associated with increased infiltration of stromal CD68^+^ macrophage in the TME of the high-risk group (60). Studies on breast cancer cell lines, such as BCC and MCF-7, showed that these cells provide their required energy via degrading BCAAs and can generate mevalonate by leucine degradation. Additionally, metabolic flux analysis around the mevalonate node demonstrated that high concentrations of acetoacetate were produced from BCAA-derived carbon, and this metabolite could be a potential source for lipid synthesis (61). These data indicate that the degradation of BCAAs could be a potential carbon/energy source for tumor cell proliferation and a potential therapeutic target for cancer treatment.

Unexpectedly, an investigation found that the increased levels of BCAA, by increasing dietary intake in mice or the genetic model, inhibited tumor growth and breast cancer lung metastasis. According to the survival analysis results, the expression of genes involved in BCAA catabolism, such as PPM1K, is robustly correlated to tumorigenesis in patients with breast cancer. Knockout of *ppm1k* in mice led to the accumulation of BCAAs due to impaired BCAA catabolism, increasing infiltrated NK cells and inhibiting tumor growth in the studied animals without affecting tumor cell proliferation and vasculature (11).

Metabolic diseases, such as type 2 diabetes mellitus and obesity, could be associated with human malignancies, and adipose tissue is actively involved in developing these disorders. Based on available knowledge, white adipose tissue stores excess energy, whereas brown and beige adipose tissues (thermogenic fats) can convert energy to heat and act as metabolic sinks of glucose, amino acids, and fatty acids (62). In the TME, these activated thermogenic adipocytes induce cancer development by providing fuel sources and leading to chemoresistance (63). Therefore, there seems to be a close relationship between adipose tissue and BCAA catabolism, which can affect tumor metabolism and lead to cancer development (64). Angiopoietin-like-4 (ANGPTL4) regulates lipids and BCAAs metabolism and is involved in tumor metastasis and progression. A study shows that ANGPTL4 is downregulated in osteosarcoma (OS). There is a negative correlation between the ANGPTL4 expression and BCAA catabolism in OS cell lines and human samples. Therefore, *ANGPTL4* knockdown in OS cells leads to accumulating BCAAs, activating the mTOR pathway, and boosting OS cell proliferation and progression. Accordingly, targeting the ANGPTL4/BCAA/mTOR axis could be a potential therapeutic target to reduce OS development (65).

It has been discovered that BCAT1 is overexpressed at the protein level in tumor cells and tissues isolated from patients with metastatic lung cancer. Transcriptomic data analysis in the TCGA database also demonstrated that upregulation of *BCAT1* transcription was associated with poor overall survival. This is because high concentrations of BCAT1 depleted α-KG and increased *SOX2* expression. Since *SOX2* is a transcription factor in regulating stemness and metastasis of cancer cells, upregulation of BACT1 can induce tumor development (66).

In human blood malignancies, a study reported that BCAA metabolism affects human leukemia cells to preserve their stemness regardless of lymphoid or myeloid types. Analysis of human primary acute leukemic cells showed that these cells could express BCAA transporters for BCAA uptake, BCAT1, and had higher levels of cellular BCAA as well as α-KG than CD34^+^ hematopoietic stem progenitor cells (HSPCs) as control (67). In xenograft leukemia models, BCAA deprivation from the daily diet significantly suppressed tumor cells’ self-renewal, expansion, and engraftment. Moreover, suppressing BCAA catabolism in primary ALL or AML cells lead to the inactivation of the polycomb repressive complex 2 (PRC2) function. PRC2 regulates stem cell signatures by preventing embryonic ectoderm development (EED) and zeste homolog 2 (EZH2) transcriptions as PRC components (67).

Considering the dual role of BCAAs in inhibiting the growth of tumor cells and tumor development, it appears that targeting different components involved in the catabolic pathways of BCAAs can be a merit in cancer therapy.

## 5 Therapeutic interventions

BCAAs play an essential role in the metabolism and biogenesis of macromolecules, and as discussed, their catabolism can affect the metabolism and behavior of tumor cells through different mechanisms. This section discusses the BCAA-related therapeutic intervention for treating various human malignancies (**Table 1**).

**Table 1:** BCAA-related therapeutic approaches for the treatment of cancer.

	Approach/drug	Type of cancer	Outcomes	Ref
***Targeted Therapies***	BCAT1 knockdown	Malignant melanoma Human study	• Inhibiting the proliferation and migration of melanoma cells and decreasing oxidative phosphorylation	(68)
	BT2	HCC *In vitro*	• Increasing the residuals of BCKDH in culture medium • Inhibiting BCKDK	(55)
	Silencing of BCKDK + doxorubicin	TNBC *In vitro*	• Downregulating BCKDK expression • Reducing the intracellular concentrations of BCKAs • Reducing the expression of genes involved in mitochondrial metabolism and electron complex protein • Consuming oxygen and ATP generation • Intensifying apoptosis and caspase activity • Inhibiting the proliferation of TNBC cells • Upregulating sestrin 2 and simultaneously decreasing mTORC1 signaling and protein synthesis	(69)
	BCKDK inhibition by siRNA + paclitaxel	Breast and ovarian cancer cells *In vitro*	• Decreasing BCAA levels • Inducing the antitumor effects of paclitaxel • Deactivating the mTORC1-Aurora pathway	(70)
	BCKDK knockdown with shRNAs	Ovarian cancer *In vivo* and *ex vivo*	• Repressing ovarian cancer cell proliferation and migration	(71)
	Transfection of anti-*BCKDK* siRNA	Non-small cell lung cancer *In vitro*	• Reducing the expression of BCKDK • Decreasing BCKDE1α phosphorylation • Inhibiting the proliferation of the mentioned tumor cells • G0/G1 cell cycle arrest • Upregulating P21	(72)
***Dietary interventions***	Rich-BCAA diet	Pancreatic intraepithelial neoplasia *LSL-Kras^G12D/+^*; *Pdx1-Cre* (KC) mice	• Inducing tumor progression	(73)
	Rich-BCAA diet	Pancreatic cancer Case-control study	• A positive association between dietary BCAA intake and the risk of pancreatic cancer	(74)
	BCAA supplementation	HCC Human study	• Improving npRQ • Increasing albumin levels • Improving the quality of life • Reducing the Child-Pugh score • Decreasing the recurrence rate • Prolonging the overall survival	(8)
	High-BCAA diets	Breast cancer *In vitro/In vivo*	• Suppressing the growth of breast cancer cells and related lung metastases in mice • Impairing tumor cell migration and invasion • Downregulating N-cadherin	(11)
	High-BCAA diets	Postmenopausal breast cancer Human study	• Decreased risk of Postmenopausal breast cancer	(75)
	High-BCAA diets	CRC A large case-control study	• An inverse association between BCAA intake and the risk of sigmoid colon cancer risk	(76)
	High-BCAA diets	CRC Human study	• A positive association between a higher dietary BCAAs intake and the risk of all-cause mortality in CRC patients	(77)

### 5.1 Targeted therapies

Evidence revealed that modulating the accumulation of BCAAs can regulate tumor cell proliferation, tumor burden, and overall survival. Dietary BCAA intake is also associated with cancer development in some malignancies. Moreover, reducing BCAA catabolism via therapeutic tumor interventions could have functional benefits (48). Based on the available studies, the enzymes involved in the catabolism of BCAAs, such as BCAT1, BCAT2, and BCKDK, could be potential therapeutic targets for cancer treatment (35, 78). However, some studies reported that BCAT1 and BCAT2 are not involved in human cancers such as PDAC because these enzymes can not induce PDAC tumor formation.

Consequently, the origin tissue is a critical factor in how tumors meet their metabolic needs (79). Based on these findings, perhaps the inhibition of these enzymes may not be effective in some cancers. Nonetheless, analysis of samples from patients with malignant melanoma and mouse malignant B16 melanoma cell lines showed that BCAT1 was overexpressed in both sample types. Moreover, BCAT1 knockdown inhibited the proliferation and migration of melanoma cells and decreased oxidative phosphorylation (68). These findings show that BCAT1 can be a suitable therapeutic target in treating human cancers such as malignant melanoma. It has been revealed that the cytosolic BCAT isoform could be associated with human epidermal growth factor receptor 2 (HER2)-positive tumors, while its mitochondrial isoform is highly expressed in estrogen (ER)-positive tumors. Therefore, targeting BCAT enzymes should consider their isoforms in various cancers with different phenotypes (80).

Evidence has shown that targeting BCKDK with small-molecule and allosteric inhibitors could be effective in suppressing tumor cell development. The mechanism of action of these inhibitors is binding to BCKDK, prompting movements within the N-terminal domain helix, and finally detaching BCKDK from BCKDH and degrading BCKDK (55, 81). (S)-α-chlorophenylpropionate and 3,6-dichloro-1-benzothiophene-2-carboxylic acid (BT2) are BCKDK allosteric inhibitors (82, 83). However, BT2 is more effective than (S)-α-chlorophenylpropionate, and has greater metabolic stability. After treatment with BT2, BCKDH activities increased in the culture medium of primary hepatocytes (55). BCKDK also activates the RAS/RAF/MEK/ERK pathway by phosphorylating MEK, which promotes tumor cell proliferation (54). It has been reported that, like genetic and pharmacological inhibition of BCKDK, treating triple-negative breast cancer (TNBC) cells with doxorubicin downregulated BCKDK expression, reducing the intracellular concentrations of BCKAs. Additionally, silencing of BCKDK in TNBC cells reduced the expression of genes involved in mitochondrial metabolism and complex electron protein, as well as the consumption of oxygen and ATP generation. BCKDK silencing also induced apoptotic pathways. Silencing BCKDK in combination with doxorubicin intensified apoptosis and caspase activity, inhibiting TNBC cell proliferation. Suppressing BCKDK in TNBC cells could also upregulate sestrin 2 while simultaneously decreasing mTORC1 signaling and protein synthesis. Thus, BCAAs flux remodeling by BCKDK inhibition in TNBC could be a potential therapeutic approach to hinder tumor growth and progression (69). Another investigation showed that inhibition of BCKDK by siRNA or chemical inhibitors could reduce BCAA levels and synergistically induce the antitumor effects of paclitaxel in breast and ovarian cancer cells. However, BCKDK inhibition also deactivated the mTORC1-Aurora pathway, overcoming M phase cell cycle arrest stimulated by paclitaxel (70). In patients with ovarian cancer, a higher level of BCKDK is associated with advanced pathological grades. Moreover, BCKDK overexpression promotes ovarian cancer cell proliferation and migration by inducing the MEK/ERK pathway. Accordingly, BCKDK knockdown with shRNAs repressed ovarian cancer cell proliferation and migration *in vivo* and *ex vivo* (71). Transfection of anti-*BCKDK* siRNA into non-small cell lung cancer cells, including A549, HCC827, and H1299, reduced the expression of BCKDK, decreased BCKDE1α phosphorylation, and inhibited the proliferation of tumor cells in these cancers. In addition, G0/G1 cell cycle arrest and an increase in the expression of P21 occurred following the inhibition of BCKDK (72). Ubiquitin-specific protease 1 (USP1), a human deubiquitinase, plays a significant role in regulating the cellular responses to DNA damage and controlling cell differentiation (84). BCAAs promote the expression of USP1 protein, and USP1 can deubiquitylate BCAT2. Furthermore, suppressing USP1 can inhibit tumor cell growth and proliferation as well as clone formation in PDAC mice models. These outcomes indicate that targeting USP1/BCAT2 in the BCAA metabolic pathway could be a therapeutic strategy for treating PDAC (73).

### 5.2 Dietary interventions

The results of studies on different cancers show several contradictions regarding the dietary intake of BCAAs as a therapeutic approach. For instance, an experimental study on *LSL-Kras^G12D/+^*; *Pdx1-Cre* (KC) mice revealed that a BCAA-rich diet stimulates the progression of pancreatic intraepithelial neoplasia (73). A multicentric Italian case-control study also reported a positive association between dietary intake of BCAAs and the risk of pancreatic cancer (74). Supplementation of BCAAs to 1594 HCC patients undergoing locoregional therapies, including radiofrequency ablation, hepatic artery infusion chemotherapy, or transarterial chemoembolization, could improve the non-protein respiratory quotient (npRQ, which represents the ratio of carbohydrate to fat oxidation), increase albumin levels, and improve quality of life. Furthermore, BCAA supplementation reduced the Child-Pugh score (a score used to evaluate the prognosis of chronic liver disease), decreased the recurrence rate, and prolonged overall survival (8). It is possible that supplement therapy with BCAAs can help reduce the side effects caused by conventional cancer therapies, such as chemotherapy, radiotherapy, or surgery. In this regard, an investigation revealed that administering BCAAs during the oncological surgical period led to satisfactory outcomes in reducing post-operative morbidity from ascites and infections (85). However, it should be noted that the catabolism of this group of amino acids may potentially benefit tumor growth, and further studies in this field are needed to determine the optimal dosage as well as the specific types of cancer.

On the other hand, it has been reported that high-BCAA diets can suppress the growth of breast cancer cells and lung metastases in mice. A high concentration of BCAAs in the culture medium impaired the ability of breast cancer cells to migrate and invade, likely due to the downregulation of N-cadherin. Additionally, a low BCAA diet increased the colonization of tumor cells in the lung. These data revealed that high BCAA diets effective in treating breast cancer by suppressing tumor cell growth and metastasis (11). Another study also found a significant association between higher dietary intake of BCAAs and a decreased risk of postmenopausal breast cancer (75). However, the evaluation of the association between long-term dietary intakes of BCAAs and invasive breast cancer risk showed no association between dietary intakes of total or individual BCAAs and the risk of breast cancer (86).

A large case-control study on colorectal cancer patients showed an inverse association between BCAA intake and the risk of sigmoid colon cancer risk (76). These data are consistent with findings from several large US cohorts that do not support the hypothesis of a positive association between dietary BCAA intake and colorectal cancer risk (78). Another investigation suggested positive associations between higher dietary intake of BCAAs and the risk of all-cause mortality in CRC patients (77). Considering the contradictions in this area, further studies are needed to confirm these results, identify underlying mechanisms, and caution should be exercised when supplementing BCAAs in individuals with cancer (87).

## 6 Concluding remarks

In conclusion, extensive research has established the undeniable role of BCAAs and the activated biological pathways they engage in cancer. Activation of various enzymes in the catabolism of BCAAs leads to the production of metabolites that indirectly contribute to tumor growth and development. Therefore, targeting these enzymes presents a promising therapeutic approach for cancer treatment. However, the effectiveness of such interventions varies across different cancers due to the presence of diverse isoforms of these enzymes in distinct cancer phenotypes, posing challenges in treatment.

Contrastingly, supplement therapy with BCAAs has demonstrated tumor growth inhibition in certain cancers while showing no impact on others. Consequently, it is essential to consider the contradictory data derived from different studies and acknowledge the presence of numerous confounding factors that influence the results. To obtain reliable and valid outcomes, it is imperative to conduct studies with larger sample sizes and employ rigorous measures to minimize the impact of confounding factors. By doing so, we can comprehensively understand the complex relationship between BCAAs, cancer, and potential therapeutic interventions.

## Author contributions

KJ: Conception, design, and inviting co-authors to participate. EX, BJ and YC: Writing original manuscript draft. KJ and YC: Review and editing of manuscript critically for important intellectual content and provided comments and feedback for the scientific contents of the manuscript. All authors contributed to the article and approved the submitted version.
